# The Role of Heparin in Embryo Implantation in Women with Recurrent Implantation Failure in the Cycles of Assisted Reproductive Techniques (Without History of Thrombophilia) 

**Published:** 2015-06

**Authors:** Kobra Hamdi, Shahla Danaii, La’ya Farzadi, Sedigheh Abdollahi, Allahverdi Chalabizadeh, Somayae Abdollahi Sabet

**Affiliations:** Women's Reproductive Health Research Center, Tabriz University of Medical Sciences, Tabriz, Iran

**Keywords:** Heparin, Recurrent implantation failure, ART, Pregnancy test

## Abstract

**Objective:** Several studies have shown the improving effect of heparin on the outcomes of ART. Moreover, it has been reported that adding heparin in non-thrombophilia patients with RIF is useful.The aim of this study was to evaluate the beneficial effects of heparin on ART outcomes in women with history of recurrent implantation failure (RIF) and without history of congenital or acquired thrombophilia in a randomized, controlled clinical trial (RCT).

**Materials and methods:** In this study, 100 patients with a history of two or more failures in implantation in cycles of ART were randomly subdivided into two groups of study and control. Patients of the control group just received the luteal phase support. In the patients of study group, in addition to the routine support of luteal phase following in vitro fertilization (IVF) or intra cytoplasmic sperm injection (ICSI), 5000 units of subcutaneous heparin was administered for 15 days from the day of oocyte pick up. Pregnancy test (β-HCG) was done for patient of two groups 15 days after IVF.

**Results:** In the study group, pregnancy test was positive in 16 (32%) patients and negative in 34 (68%) patients. In the control group, pregnancy test was positive in 15 (30%) patients and negative in 35 (70%) patients. There was no significant difference between two groups for the role of heparin in the pregnancy rate (p = 0.5).

**Conclusion:** Although the effect of heparin on pregnancy was not statistically significant in this study, with regard to the numerous benefits of this agent, it is recommended to study its effects in further studies with lager sample size.

## Introduction

Since the beginning of assisted reproduction treatment (ART) in 1978, great progresses have emerged in the techniques ofovulation stimulation, the production of oocytes, micro injection, embryo production and transfer; however, implantation rate continues to be low, that is the main factor in decrease of live birth rate in ART cycles (1). Implantation of embryo is a complex process and depends on many variables thatmost of themhave not been defined yet completely.Implantation requires coordination between multiple events such as the evolution of the trophoblast and the timing someof molecules that play an important role in apposition, penetration and invasion of embryo to the endometrium (2). Despite transfer of embryos with good quality, implantation failure is a relatively common event which leads to couples disappointment and physician discouragement. Failure of implantation has been attributed to several factors, but no specific relationship has been established in most of cases so far (2). Several studies have pointed out the effect of heparin on improving the outcomes of assisted reproductive techniques (ART). Greatest impact of heparin has been seen in thrombophilia patients, especially inpatients with anti-phospholipid antibodies.It has also been useful to add heparin in non-thrombophillic patients with recurrent implantation failure (RIF) (3).

In addition to theanticoagulant effect of the heparin, its anti-inflammatory role has been the subject of many investigations. In animal models, it has been shown thatheparin disaccharidesinhibit TNF-α producedby macrophage, so inflammationrelated to immune systemcouldbe reduced.Some factors associated with heparin such asHSPG, (heparin sulfate proteoglycans) heparin-binding EGF-like growth factor (HB-EGF) may have a role in reproductive functions such as blastocyst attachment to the uterine epithelium, blastocyst invading and growth stimulation of syncytial trophoblast cells (4). Other effects of heparin include the anti-apoptotic effect causingapoptosis inhibition of trophoblast in first trimester by activating the survival pathway of growth factor receptor and blocking the trophoblast apoptosisthat is mediated by αPL (α-poly-L-lysine). It is shown that heparin regulates the Adhesion, apoptosis and cell-to-cell interactions during implantation. The heparin enhances trophoblast invasion through matrix metalloproteinase (MMP), With regard to mentioned roles of heparin, it might be concluded that heparin also plays roles in fetal implantation in addition to anticoagulation effect (5).

Based on the available information about heparin and its role in implantation, a retrospectiveclinical trial (RCT) was designed in order to achieve the beneficial effects of heparin on ART outcomes in women with recurrent implantation failure (RIF) (2 times or more) with no history of hereditary or acquired thrombophilia.

## Materials and methods

In a randomized, controlled clinical trial (RCT) in from January 2012 to December 2013 in the infertility Center of Al_Zahra Hospital, Tabriz, Iran a number of 100 infertile couples with infertility and history of RIF were selected and assigned into two groups of study and control randomly.

***Inclusion criteria***

1.The history of at least 2 cases of implantation failurewith fresh embryo with good grades (A-B).

     2.The women’s age lower than 40 years.

     3.Fresh ejaculated sperms for IVF or ICSI (intracytoplasmic sperm injection) 

     4.no history of hormonal, coagulation and immunologic disorders

     5.Normal uterine cavity (in HSG or hysteroscopy)

***Exclusion criteria***

1.Women with a medical history of anticoagulation therapy.

     2.Obvious causes of implantation failure (hydrosalpinx, submucosal myoma, the absence of embryos with A or B grade for transfer)

     3.Clinical findings or laboratory evidence of hereditary or acquired thrombophilia.

     Initial evaluation of patients included HSG, hysteroscopy, ovarian reserve tests (FSH, LH and estradiol on the third day of menstruation) coagulation tests as well asgenetic analysis of mutations in three genes for factor V (factor V Leiden), methylene-tetrahydro folate reductase (MTHFR) gene and the prothrombin or factor II. Furthermore, because of the possibility of Heparin Induced thrombocytopenia, routine blood tests, including the determination of the platelet count was performed in these patients and those with normal platelet counts were enrolled. Ovulation stimulation were the same in both groups and therapeutic cycles were done for all patients and embryo transfer cycles started in patientswith embryoswith grades A and B. In equal conditions, patients were divided into two groups of control and study. Fifty people regarded as control just received the progesterone for luteal phase support. In the patients of study group, in addition to the routine support of luteal phase following in vitro fertilization (IVF) or intracytoplasmic sperm injection (ICSI), 5000 units of subcutaneous heparin were administered for 15 days from the day of oocyte pick up. Pregnancy test (β-HCG) was done for each patient of two groups 15 days after IVF. The results of pregnancy were com pared between two groups. 

***Ethical considerations***

After obtaining written consent from all patients of two groups and explanationabout the role of adjuvant heparin, procedure was begun. Since heparin was at low prophylaxis dose, no side effects were seen. All information has been kept completely confidential.

***Data analysis***

At the end of study, all the information from patients was statistically analyzed using SPSS version 16. Thereafter, all data were provided in the statistical descriptive methods (mean± SD), frequency and percent. Analysis and comparison of the qualitative, qualitative-quantitative and quantitative variables were done using Chi-Square, Student t-test and difference tests respectively. The p value was considered significant in less than 0.05.

## Results

**Figure1:**
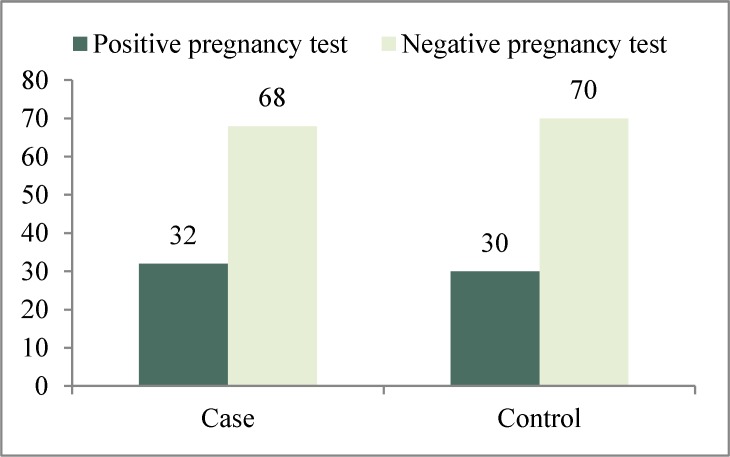
Comparison of pregnancy rate between two groups

From 100 patients selected, 50 patients were in the study group (receiving 5000 U/day SQ (subcutaneous) heparin from the day of oocyte pick up and 50 were in control group (just luteal phase support, no heparin).The mean age of patients in thestudy group was 32.46 ± 5.14 years (min = 22, max = 40) and in control group was 30.9 ± 4.71 (min = 24, max = 40). There was no significant difference between two groups in the age of patients (p = 0.53). At the end of the 15^th ^day, the serum β-HCG of all patients were measured that were positive in 16 (32%) patients and negative in 34 (68%) patients of study group. In the control group, beta HCG was positive in 15 (30%) and negative in 35 (70%) of patients. The difference in the rate of pregnancy in two groups was not statistically significant (p = 0.5) (Figure 1).

The mean infertility period in the study group was 7.38 ± 4.9 (min = 2, max = 16) years and 8.22 ± 5.85 (min = 1, max = 20) years in the control group with no statistically significant difference between two groups (p = 0.33).

In the study group, the embryo transfer was easy with tenaculum in 4 patients (8%), easy without tenaculum in 41(82%) patients, difficult with tenaculum in 3(6%) patients and difficult due to other instruments in 2 patients. In the control group, transfer was easy with tenaculum in 8 (16%) patients, easy without tenaculum in 40 (80%) patients, difficult with tenaculum in 1 (2%) patient and difficult due to other instruments in 1 (2%) patient with no statistically significant difference between two groups (p = 0.43).

In the study group, cervical mucus was negative in 43 (86%) and positive in 7 (14%) of patients; in the control group, it was negative in 40 (80%) and positive in 10 (20%) patients. There was no significant difference between two groups for mucus (p = 0.2).

The uterine position was in midline in 35 (70%) patients, retroverted in 6 (12%) patients and anteverted in 9 (18%) patients of study group. In the control group, it was in midline position in 34 (68%), retroverted in 10 (20%) patients and anteverted in 6 (12%) of patients. The difference in uterine position was not significant between two groups (p = 0.4).

In the study group, the thickness of endometrium on the day of HCG injection was 8-10 mm in 43 (86%) patients and 10- 12 mm in 7 (14%) patients. In the control group, in 39 (78%) patients was 8- 10 mm and in 11 (22%) patients was 10- 12 mm. The difference in endometrial thickness was not significant (p = 0.21). Endometrial pattern on the day of HCG injection was tri-line in 49 (98%) of patients and echogenic in 1 (2%) patient in the study group. The pattern was tri-line in 50 (100%) of patients in control group.

The embryo grade in study group was A in 31 (62%) patients, B in 19 (38%) patients. The embryo grade in control group was A in 34 (68%) patients, B in 16 (32%) patients. There was no statistically significant difference between two groups.

In the study group, transfer was done in 2 cm distance of fundus in 17 (34%) patients, 1.5 cm of fundus in 29 (58%) patients and in 1 cm of fundus in 4 (8%) patients. In the control group, transfer was done in 2 cm distance of fundus in 22 (44%) patients, 1.5 cm of fundus in 26 (52%) patients and in 1 cm of 

**Figure 2:**
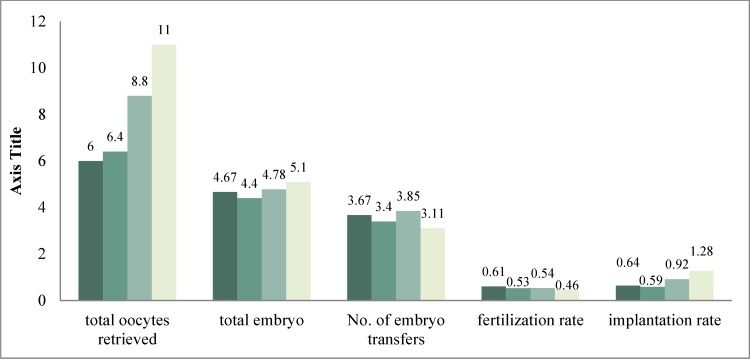
Comparison of the number of oocytes, embryos, fertilization rate and implantation rate between the study and control groups

fundus in 2 (4%) patients. There was no statistically significant difference between two groups.

The mean number of oocytes in the study group was 6.25 ± 2.65 (min = 4, max = 10) and 7.33 ± 3.2 (min = 2, max = 12) in the control group (p = 0.07) (Figure 2).

**Figure 3:**
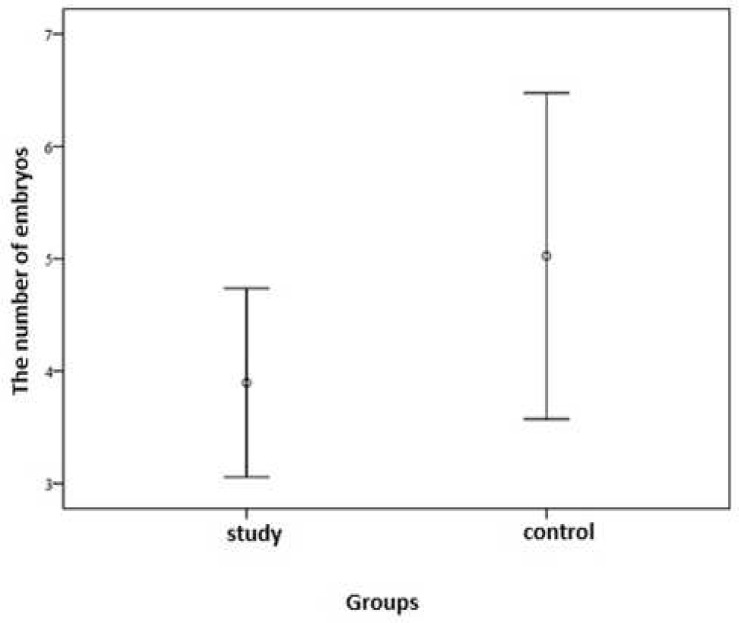
Comparison of the number of embryos between the study and control groups

The mean number of embryos in the study group was 3.5± 1.6 (min = 2, max = 7) and 5.3± 2.5 (min = 3, max = 9) in the control group (p = 0.02) (Figure 3).

The luteal phase support in the study group was progesterone ampoule in 42 (84%), cyclogest suppository in 7 (14%) patients and progesterone ampoule plus cyclogest suppository in 1 (2%) patient, this support was by progesterone ampoule in 39 (78%) patients, cyclogest suppository in 10 (20%) patients and progesterone ampoule plus cyclogest suppository in 1 (2%) patients in the control group. There was no statistically significant difference between two groups (p = 0.4).

The mean FSH level in the study group was 8.17 ± 1.6 mIU/l (min = 5.5, max = 11) and in the control group 7.38± 1.7 mIU/l (min = 5, max = 12). There were no statistically significant difference between two groups in FSH level (p = 0.65).

The mean LH levelin the study group was 7.53 ± 1.5mIU/l (min = 4, max = 9) and in control group 6.76± 1.68 mIU/l (min = 4, max = 10). There were no statistically significant difference between two groups in LH level (p = 0.58).

The mean Estradiol levels on the third day of menstruation in the study group was 90.58 ± 20.42 pg/ml (min = 54, max = 120) and in the control group 72.73 ± 19.53 pg/ml (min = 42, max = 120). There were no statistically significant difference between two groups in Estradiol level (p = 0.9).

## Discussion

Nowadays the treatment process of patients with recurrent pregnancy failure related to anti-phospholipid antibodies (APAs) or other thrombophillic disorders is focused on use of antithrombotic drugs and has led to recommendation of such antithrombolytic therapies in patients with RIFand without thrombophillic disorders. Various studies have been conducted in this area (6).

In the present study a positive pregnancy test by serum βHCG level on day 15 after embryo transfer was considered as the main outcome measure of this study. The difference in pregnancy rate in two groups of pregnant women treated with heparin and control was not statistically significant (p = 0.5), but in the heparin group was higher (32% vs. 30%), which maybe clinically significant and this difference will be increasedin studies with larger number of samples. Regardless of the successful completion of the pregnancy, heparin maybe efficient in improving implantation rates.

In the study of Urman et al, in Turkey in 2009, the effect of treatment with LMWH in patients with non thrombophilic recurrent implantation failure was studied on 150 patients subdivided into two groups of 75; according to results of this study, although it was not seen the non-significant difference, a slight increase in implantation was seen in LMWH group which was similar to our study (7).

In another study by Muhammad A. Akhtarin 2013 on the effects of aspirin and heparin as adjunctive therapy on 196 patients undergoing IVF treatment with a history of at least one implantation failure, no benefits in improving the rate of pregnancy and live births were observed (8). In another study by Ivo Nociet al. in 2011 on the effects of LMVH on non thrombophilic young women under treatment of IVF, no significant effect on increase in implantation and the rate of live birth was observed which was compatible with our study (9).

Sternet al. also observed that heparin and aspirin could not improve the pregnancy and implantation rates in the patient positive for anti-phospholipid antibodies (APAs) and positive for antinuclear antibodies (ANA). The results of our study are consistent with this study (12); however, since the patients with congenital or acquired thrombophilia were excludedin our study, this conclusion is not completely assured (10).

Sher et al, reported no improvement in the pregnancy outcome in APA-negative patients that was similar to our study, but the outcome was better in APA-positive patients (11). In most previous studies, similarly heparin improved pregnancy outcome in the majority of APA-positive cases but was without beneficial effect in APA-negative cases (11).

The most of previous studies which support the heparin efficacy in patients with IVF failure were done on APAs patients or patients with positive immunologic factors but fewer studies were done on heparin effect on IVF failure with unknown cause (12).

In our study the mean number of transferred embryos in the study group was 3.5 ± 2.26 and in the control group was 4.67 ± 1.63, but the 2% higher pregnancy rate in the study group with regard to the low number of transferred embryos (even in the presence of a non-significant p value) is important and may be increased by increasing the sample size and the follow-up time of patients.

There was no significant difference between heparin (study) and control group in pregnancy outcome; however, the mean number of embryos, oocytes and the mean number of transferred embryos was higher in control group which shows higher efficacy in heparin group.

In the previous studies about the heparin effect on patients with immunologic disorders, APAs patients have been mostly studied and the efficacy of heparin has been confirmed. In our study, there was no APA-positive cases, so we cannot make a comparison, but in patients without history of anticoagulant therapy, no obvious cause of implantation failure (hydrosalpinx, submucosal myoma, absence of embryos with A, B grades and no clinical and laboratory findings of congenital or acquired thrombophilia) improvement of the pregnancy outcome was not seen.

In the study of Feinmanet al., heparin and aspirin significantly increased live birth rate in patients with recurrent implantation failure and positive APA as compared with the untreated patients. In this study, the pregnancy rate in APA-negative women treated with aspirin and heparin was significantly higher than the untreated women (13), but in the present study, the increase in pregnancy rate in patients with idiopathic RIF treated with heparin was not observed.

According to the results, the effect of heparin on pregnancy was not statistically significant in this study. However, with regard to the higher rate of pregnancy in the group under treatment with heparin but its insignificant difference, more studies with more cases appear to be necessary. Moreover, the exact study of reasons of recurrent implantation failure of IVF because of limited cases of this studywas not possible so more multi-central studies seem to be essential.
